# Depression diagnoses following the identification of bipolar disorder: costly incongruent diagnoses

**DOI:** 10.1186/1471-244X-10-39

**Published:** 2010-06-04

**Authors:** Michael D Stensland, Jennifer F Schultz, Jennifer R Frytak

**Affiliations:** 1 Agile Outcomes Research, Inc, Rochester, Minnesota, USA; 2 University of Minnesota, Department of Economics, Labovitz School of Business and Economics, Duluth, Minnesota, USA; 3 i3 Innovus, Eden Prairie, Minnesota, USA

## Abstract

**Background:**

Previous research has documented that the symptoms of bipolar disorder are often mistaken for unipolar depression prior to a patient's first bipolar diagnosis. The assumption has been that once a patient receives a bipolar diagnosis they will no longer be given a misdiagnosis of depression. The objectives of this study were 1) to assess the rate of subsequent unipolar depression diagnosis in individuals with a history of bipolar disorder and 2) to assess the increased cost associated with this potential misdiagnosis.

**Methods:**

This study utilized a retrospective cohort design using administrative claims data from 2002 and 2003. Patient inclusion criteria for the study were 1) at least 2 bipolar diagnoses in 2002, 2) continuous enrollment during 2002 and 2003, 3) a pharmacy benefit, and 4) age 18 to 64. Patients with at least 2 unipolar depression diagnoses in 2003 were categorized as having an incongruent diagnosis of unipolar depression. We used propensity scoring to control for selection bias. Utilization was evaluated using negative binomial models. We evaluated cost differences between patient cohorts using generalized linear models.

**Results:**

Of the 7981 patients who met all inclusion criteria for the analysis, 17.5% (1400) had an incongruent depression diagnosis (IDD). After controlling for background differences, individuals who received an IDD had higher rates of inpatient and outpatient psychiatric utilization and cost, on average, an additional $1641 per year compared to individuals without an IDD.

**Conclusions:**

A strikingly high proportion of bipolar patients are given the differential diagnosis of unipolar depression *after *being identified as having bipolar disorder. Individuals with an IDD had increased acute psychiatric care services, suggesting higher levels of relapses, and were at risk for inappropriate treatment, as antidepressant therapy without a concomitant mood-stabilizing medication is contraindicated in bipolar disorder. Further prospective research is needed to validate the findings from this retrospective administrative claims-based analysis.

## Background

Bipolar disorder, a severe and recurrent mental disorder, is characterized by episodes of elated and depressed mood. Epidemiological studies have reported lifetime prevalence ranging from 0.8% - 5.1% [1-3]. However, in most private claims databases, the prevalence of treated bipolar disorder has been found to be lower (0.2%) [4,5]. This discrepancy can be attributed to 2 factors: Only 40% of individuals with bipolar disorder have private insurance [6], and many patients are not correctly diagnosed.

The results of screening studies for bipolar disorder have shown that a strikingly high proportion of individuals seeking treatment for symptoms of bipolar disorder are not diagnosed. In a recent primary care screening study, less than 10% of individuals who screened positive for bipolar disorder on a brief screening tool (Mood Disorders Questionnaire; MDQ) reported being previously diagnosed with bipolar disorder [7]. In another study that rigorously confirmed the bipolar diagnosis, 25.6% of psychiatric outpatients with bipolar I and 50.5% with bipolar II disorder were not diagnosed [8]. Other survey research found an average time lag between onset of symptoms and diagnosis of 7-10 years [6,8].

Part of the challenge of recognizing bipolar disorder is differentiating it from other disorders, particularly non-bipolar, or unipolar, depression [9], given the high degree of symptom overlap. The symptoms a bipolar patient experiences during a depressive episode meet the diagnostic criteria for major depressive disorder; the disorders are differentiated based on the patient's history of manic or hypomanic symptoms [10]. Unfortunately, patients often do not recall past manic symptoms or do not recall them as problematic [11]. Further, depressive symptoms are present 3 times as often as manic symptoms in patients with bipolar disorder [12]. Thus, eliciting a history of manic or hypomanic symptoms is a difficult challenge for clinicians. Yet, when such a history remains unknown, patients are likely to receive a unipolar depression diagnosis and treatment that is inappropriate or contraindicated for bipolar disorder, such as antidepressant monotherapy and lack of appropriate mood-stabilizing medication.

Because of the important treatment implications of this differential diagnosis, efforts have been made to improve initial identification of bipolar disorder and differentiate it from unipolar depression. Review articles have described the subtle clinical characteristics that differentiate not-yet-recognized bipolar disorder from unipolar depression [13,14]. In addition, screening tools for bipolar disorder, such as the MDQ [15] and a claims-based screening algorithm [16], have been developed to help identify unrecognized bipolar disorder.

These efforts assumed that an accurate diagnosis of bipolar disorder, once achieved, would remain with the patient throughout future treatment, but our previous research suggests that an initial diagnosis of bipolar disorder may be less stable than previously thought. We found that 27.5% of individuals initially diagnosed with bipolar disorder received unipolar depression disorder diagnoses *after *they had been diagnosed with bipolar disorder [17,18]. Those patients who had received incongruent depression diagnoses (IDDs) had an 82% increase in mental health hospitalizations, a 147% increase in mental health emergency room (ER) visits, and an 80% increase in mental health ambulatory visits, resulting in an increase of $3189 per patient per year in treatment costs relative to those patients who were not given the incongruent unipolar depression diagnosis. Analysis of provider switching revealed that the lack of continuity of care among mental health providers was the most convincing mechanism for the *loss *of the bipolar diagnosis.

Our earlier study [18], selected a population of individuals who had been newly diagnosed with bipolar disorder in their administrative claims. However, a health management intervention study to validate those findings would be simpler to implement and potentially have larger cost savings if conducted in the larger population of all individuals with a diagnosis of bipolar disorder, rather than just those newly diagnosed. This potential intervention could start on a given date, examine all individuals with a history of a bipolar diagnosis, screen for new claims with depression diagnoses from a different healthcare provider, and then intervene to inform the provider of the previous bipolar disorder diagnosis. Identifying the best population to intervene in is of paramount concern for designing a health management intervention.

The objectives of the current study were to identify the costs of an incongruent diagnosis by expanding the study population from initially diagnosed bipolar patients to all bipolar patients. Specifically, we assessed the rate of IDDs given to individuals with a history of bipolar disorder as of January 1, 2003 and assessed the increased costs associated with the IDD. We intend to inform the design and population for a potential intervention by analyzing this study population with similar methods from our prior research.

## Methods

This study design used retrospective, longitudinal claims data from a large, national, managed-care organization providing coverage for inpatient care, ambulatory services, and prescription drugs. The study sample was derived from commercially insured health plan members or members with Medicaid managed-care coverage, 18 to 64 years of age, who had medical and pharmacy benefits, and who were continuously enrolled in the health plan from January 1, 2002 until December 31, 2003. The data were used with permission from the data source. Individuals may not have had continuous enrollment during the study period for a variety of reasons including, but not limited to, a loss of employment, a switch in employers, an employer's switching of insurance companies, failure to pay insurance premiums, discontinuation of insurance coverage, or death. Study patients were required to have a minimum of 2 bipolar diagnoses in 2002. Because we used a prevalence-based sample rather than patients newly diagnosed, the index date was set to January 1, 2003 for all patients. With the exception of the definition of the index date and the precise time period for continuous enrollment, the study methods and variable definitions mirror those of our previously published study [18].

To control for background differences between IDD and no incongruent depression diagnosis (NIDD) patients, predicted probabilities were used as a covariate in the outcome models [19]. The predicted probabilities were calculated from a backward elimination logistic regression predicting IDD status based on variables measured in the baseline period. Backward elimination was used to identify the covariates and to reduce the potential for bias from multicollinearity and endogeneity. The independent variables in the model were measured during the baseline period and are listed in Table 1.

**Table 1:** Descriptive statistics: demographic, utilization, and cost variables

	Cohort					
	NIDD (N = 6581)		IDD (N = 1400)		Uni- variate	Multivariate
Demographic Variables:					p	p
Men, n, %	2378	36.13%	391	27.93%	<.0001	
Age, mean, SD	40.62	11.02	39.73	10.97	.0064	.0317
**Region**						
Northeast, n, %	747	11.35%	213	15.21%	<.0001	
South, n, %	2781	42.26%	570	40.71%	.2879	.0845
West, n, %	860	13.07%	159	11.36%	.0816	
Midwest, n, %	2193	33.32%	458	32.71%	.6605	
**Plan Type**						
Commercial, n, %	6232	94.70%	1344	96.00%	.0437	
Medicaid, n, %	349	5.30%	56	4.00%	.0437	
Baseline Variables:						
Baseline Unipolar Dx, n, %	886	13.46%	936	66.86%	<.0001	<.0001
Number of Unipolar Dxs^a^	0.95	3.24	8.96	11.60	<.0001	<.0001
Total Costs^a^	9056.62	18371.86	13448.57	20021.63	<.0001	.0163
M H Ambulatory Cost^a^	835.59	1704.91	1999.31	4421.06	<.0001	<.0001
Non-MH Ambulatory Cost^a^	2089.39	4000.50	2835.56	5239.78	<.0001	.0652
Mental Health ER Cost^a^	48.42	304.59	96.40	525.63	.001	
Non-MH ER Cost^a^	206.93	845.65	293.98	1041.55	.0034	
MH Inpatient Cost^a^	793.66	3428.65	2141.36	7325.41	<.0001	
Non-MH Inpatient Cost^a^	1752.31	17979.46	1658.32	12441.45	.8141	
MH Medication Cost^a^	1745.42	1909.65	2153.20	2032.10	<.0001	.018
Total Medication Cost^a^	2836.37	2882.69	3545.40	3370.89	<.0001	
Number of Psychotherapy Sessions^a^	5.92	9.43	13.14	13.77	<.0001	
Antidepressant Day Supply^a^	186.58	196.25	276.30	209.07	<.0001	<.0001
Lithium Day Supply^a^	55.36	111.00	31.05	81.58	<.0001	<.0001
Benzodiazepine Day Supply^a^	64.38	123.73	98.61	144.33	<.0001	.0015
Anticonvulsants Day Supply^a^	137.94	171.75	132.97	162.30	.3035	<.0001
Antidepressant Monotherapy^b^	0.082	0.27	0.10	0.30	.031	.0163
Number of Claims w/ADHD Dx^a^	0.21	1.51	0.29	2.14	.2126	.0716
Number of Claims with Non-MH Unique Dx^a^	12.62	9.38	15.32	10.47	<.0001	.0403
Index BP Dx on Inpatient Claim, n, %	312	4.74%	163	11.64%	<.0001	<.0001
BP Diagnosis on ER Claim, n, %	273	4.15%	28	2.00%	.0001	.0225
Index BP Dx Mixed Episode, n, %	1208	18.36%	222	15.86%	.0269	.0584
Index BP Dx Unspecified Episode, n, %	3020	45.89%	574	41.00%	.0008	.0094
Charlson Comorbidity Index^a^	0.51	1.14	0.58	1.19	.042	

^a^In the 1-year period prior to index bipolar disorder diagnosis, mean, SD.

^b^Patients treated with antidepressant monotherapy were coded 1; those who were not treated with antidepressant monotherapy were coded 0, mean, SD.

NIDD = no incongruent depression diagnosis; IDD = incongruent depression diagnosis; SD = standard deviation; Dx = diagnosis; MH = mental health; ER = emergency room; ADHD = attention deficit/hyperactivity disorder; BP=bipolar

Negative binomial regression models were used to investigate the differences in the number of mental health providers, general practitioners (GPs), and other providers in the follow-up period across the 2 cohorts, controlling for baseline covariates, including the number of providers (mental health, GP, other) in the baseline period. Health care utilization was estimated using negative binomial models. Two-part models were used to analyze the relationship between IDD and health care costs. These models deal with the unique characteristics of medical expenditure data, which are typically skewed and censored. The first step was to estimate whether individuals had any medical expenditures using logistic regression. In the second step, a generalized linear model (GLM) was used to estimate positive costs. GLMs account for non-constant variance and maintain the original scale of the data, thus eliminating the need to transform cost data to achieve homoskedasticity and the need to retransform using a Duan smearing estimator for interpreting results [20]. The results of the 2-part model were combined to predict medical expenses for an individual by multiplying the prediction from each part of the model (the probability of positive expenses times the predicted medical expense from the GLM specification) [21].

To integrate the 2-part model, we first derived predicted cost estimates by running 2 prediction models: the first assuming the entire sample had an IDD and the second assuming the entire sample did not. We then calculated predicted probabilities of health care utilization. Predicted costs were combined with predicted probabilities of having any resource utilization (to account for individuals with 0 visits and 0 costs).

To specify the cost models, we used a variant of the Park test to determine the appropriate GLM distribution and link function [22]. The gamma distribution with a log-link function was used to estimate positive costs. We calculated robust standard errors using the Huber-White-type correction for the variance-covariance matrix of the parameter estimates.

The administrative claims data were statistically de-identified and compliant with the provisions of the Health Insurance Portability and Accountability Act (HIPPA) of 1996 standards. Therefore, this study did not require Institutional Review Board review.

## Results

**Figure 1:**
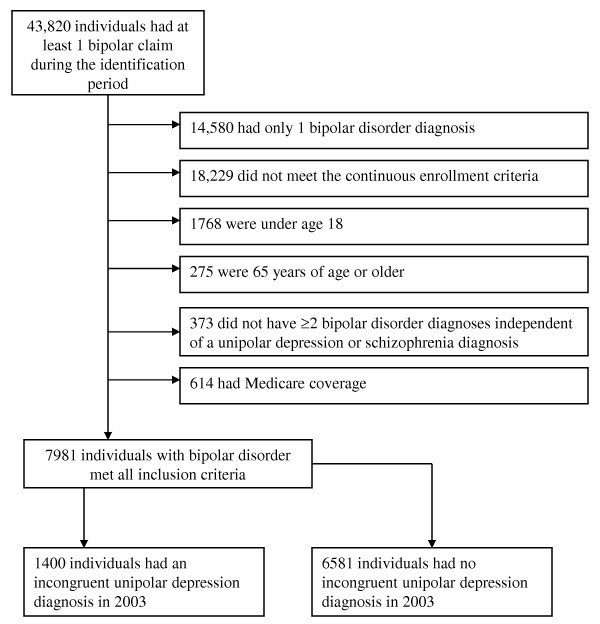
**Patient flow**.

A total of 7981 patients diagnosed with bipolar disorder met all inclusion criteria for the analysis (see Figure 1). Of these patients, 1400 (17.5%) were classified as having an IDD in the follow-up period. Approximately 66.9% (936/1400) of patients with an IDD and 13.5% (886/6581) of patients with NIDD in the follow-up period had a unipolar depression diagnosis during the baseline period. Descriptive statistics and statistical analyses of means and proportions on select variables for the 2 cohorts are shown in Table 1. A backward elimination logistic regression using the baseline variables from Table 1 to predict the likelihood of receiving an IDD in the follow-up period was relatively accurate. The area under the ROC curve indicates that the background variables were able to accurately classify a randomly selected individual according to IDD status 84% of the time. Presence of a unipolar depression diagnosis in the baseline period was a particularly strong predictor (Odds Ratio = 4.6) of a depression diagnosis in the follow-up period.

We analyzed the specialties of health care providers giving the first unipolar depression diagnosis (of the 2 required for our definition of incongruent diagnosis) to see if they differed from the specialties of health care providers who gave the first diagnosis of bipolar disorder in the baseline period. Within the IDD cohort, 1046 patients (74.1%) received their bipolar disorder diagnosis from a mental health provider. Surprisingly, an even greater number of patients (1144, 81.7%) received the first of the 2 defined unipolar depression diagnoses from mental health providers; 93 patients (6.6%) received this first unipolar diagnosis from GPs, and 163 (11.6%) from other providers (hospitals, internal medicine, emergency medicine, or unknown). In 1070 cases (76.4%), the physician giving the IDD had not previously diagnosed the patient with bipolar disorder.

The number of health care providers seen by patients in the follow-up period differed significantly between patient cohorts. IDD patients saw an average of 2.4 (standard deviation [SD] = 1.7) mental health care providers versus 1.2 (SD = 1.2) for NIDD patients (p = .001). After controlling for predicted probability and number of mental health practitioners in 2002, the number of visits with a mental health care provider was 1.83 times greater for IDD than for NIDD patients. Similar results were found for general practitioners (IDD: 1.4 ± 1.6; NIDD: 1.2 ± 1.3; p = .003, Relative risk [RR] = 1.14) and for all other practitioners (IDD: 7.8 ± 7.6; NIDD: 5.7 ± 5.9; p = .001, RR = 1.13).

IDD patients had significantly more ambulatory mental health visits, inpatient mental health visits, and ER mental health visits in the follow-up period compared to NIDD patients (see Table 2) after controlling for baseline covariates. The RRs from the models indicated that the average number of mental health ambulatory visits was 1.74 times higher for IDD patients than for NIDD patients. The mean number of mental health hospital visits and ER visits were 3.06 and 2.06 times greater, respectively, for the IDD patients. In addition, IDD patients' mental health ambulatory visits were 73% more expensive (see Table 3).

**Table 2:** Average number of visits by incongruent depression diagnosis in 2003

	Cohort			
			
	NIDD	IDD		
**Visit Type**	**Predicted Mean**	**Predicted Mean**	**RR**^**a**^	**p**

MH Ambulatory	6.57	11.46	1.74	<.0001
Non-MH Ambulatory	10.08	10.56	1.05	.22
MH Hospital	0.27	0.56	3.06	<.0001
Non-MH Hospital	0.11	0.12	1.16	.26
MH ER	0.06	0.19	2.06	<.0001
Non-MH ER	0.61	0.73	1.20	.17

^a^Relative risk of increased visits for IDD cohort relative to NIDD cohort after correcting for background differences.

NIDD = no incongruent depression diagnosis; IDD = incongruent depression diagnosis; RR = relative risk; MH = mental health; ER = emergency room

**Table 3:** Cost per patient by cohort for individuals who used the resource type

	Cohort				Cohort	
				
	IDD	NIDD			IDD	NIDD
**Visit Type**	**Predicted Mean (costs > 0)**	**Predicted Mean (costs > 0)**	**RR**^**a**^	**p**	**Probability of Resource Use**	**Probability of Resource Use**

Total Cost (N = 7900)	$10,773	$ 9132	1.18	.005		
MH Ambulatory (N = 6249)	1422	821	1.73	<.0001	0.96	0.74
Non-MH Ambulatory (N = 7332)	2462	2664	0.92	.34	0.94	0.91
MH Hospital (N = 589)	9491	7837	1.00	.97	0.18	0.05
Non-MH Hospital (N = 675)	15,704	14,841	1.06	.77	0.10	0.08
MH Emergency Room (N = 644)	658	644	1.02	.88	0.12	0.07
Non-MH Emergency Room (N = 1509)	1100	1045	1.05	.68	0.23	0.18
Mental Health Prescription (N = 7014)	2272	2299	0.99	.75	0.95	0.86
Total Prescription (N = 7679)	3522	3454	1.02	.59	0.98	0.96

^a^Relative risk of increased costs for IDD cohort relative to NIDD cohort after correcting for background differences.

Note: Generalized Linear Models with Log Link Specification

NIDD = no incongruent depression diagnoses; IDD = incongruent depression diagnoses; MH = mental health

**Figure 2:**
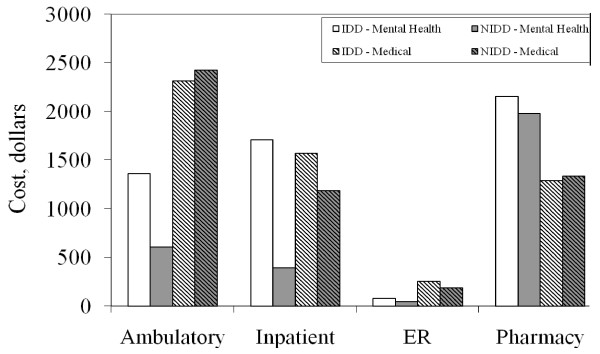
**Differences in cost components for individuals with incongruent depression diagnosis**.

Figure 2 shows the cost differences for the various components based on this integration of the 2-part model. The largest cost difference between the 2 cohorts is for inpatient mental health care ($1365 for IDD; $608 for NIDD; difference of $757). If all patients in the study received an incongruent diagnosis, average total treatment costs per person per year would be $10,773. If patients did not have the IDDs, average total treatment costs would be $9132. Thus, the treatment costs associated with an IDD were $1641 per person per year.

## Discussion

This study replicates our previous finding that a meaningful proportion of individuals with a bipolar diagnosis were given a subsequent incongruent unipolar depression diagnosis and had increased treatment costs. These results extend our previous finding from initially diagnosed to all bipolar patients. In 2003, 17.5% (1400/7981) of individuals who had been previously diagnosed with bipolar disorder were diagnosed with unipolar depression, a differential diagnosis. Diagnostic criteria indicate that once a person exhibits symptoms of mania or hypomania that person has bipolar disorder; all future depressive symptoms are part of the bipolar disorder rather than unipolar depression [10]. The IDDs were associated with a $1641 increase in treatment costs per patient, after correcting for background differences.

Given that patients were not randomized to be given a misdiagnosis of unipolar depression in a controlled study, we cannot be certain that the increased costs were due to the apparent misdiagnoses. For obvious practical and ethical reasons, one cannot complete a controlled study in which participants are randomized to be misdiagnosed. Although we corrected for background differences between the groups on January 1, 2003, we cannot be certain that the differences between groups in 2003 were due to the IDDs that occurred during 2003. The individuals with IDDs may have simply had more health care interactions in 2003 and therefore more opportunity for an incongruent diagnosis and increased costs. However, we find the misdiagnoses explanation more plausible for a variety of reasons.

The pattern of increased treatment costs is indicative of greater psychiatric relapses (see Figure 2). The 3-fold increase in rate of psychiatric hospitalization and 2-fold increase in psychiatric ER visits (see Table 2) strongly suggest that the individuals who receive the IDDs have more psychiatric relapses. The increased psychiatric outpatient and medication costs that would be expected for individuals experiencing relapses are also observed, although these costs are more difficult to interpret as they can increase for other reasons as well, such as individuals becoming more engaged in treatment.

When a patient with bipolar disorder is misdiagnosed with unipolar depression, the resulting treatment will likely be contraindicated. The primary pharmacologic treatment for unipolar depression is antidepressant monotherapy. Well-controlled clinical trials have found that antidepressant monotherapy, particularly with tricyclic antidepressants, in patients with bipolar disorder can induce mania at a higher rate than placebo [23]. Further, unipolar depression is generally not treated with mood-stabilizing medications, which represent the hallmark of treatment for bipolar disorder. Thus, appropriate pharmacologic treatment and control of symptoms depend on an accurate diagnosis of the patient's bipolar disorder.

Analysis of the number of providers and provider switching supports the notion that IDDs result, in part, from continuity of care issues as patients with this episodic and diagnostically challenging disorder interact with the health care system. Individuals receiving IDDs had twice as many mental health providers as those who did not receive IDDs. Furthermore, in 76% of the cases, the provider who gave the first incongruent unipolar depression diagnosis had not previously given the patient a bipolar diagnosis. Continuity of care may be especially important for patients with bipolar disorder, because they often do not recall manic symptoms or do not recall them as problematic [11]. Given that less than half of patients discharged after medical hospitalization are able to correctly state their diagnosis [24] and that medical records are often not received when requested [25], the physician giving the IDD may not have information concerning the patient's previous manic or hypomanic symptoms, especially when the patient is new to the physician's practice.

Interestingly, the providers making the IDD were generally mental health specialists (psychiatrists [47.7%], psychologists [12.8%], social workers [20.5%], other mental health personnel [0.71%]). We would expect that most of these individuals are well educated about the symptoms and presentation of bipolar disorder, which suggests that this rate of IDDs results from the daunting task of differentiating the 2 disorders at a given point in time rather than a lack of knowledge about bipolar disorder. In the absence of information about past manic symptoms, a diagnosis of the more prevalent unipolar depression is more reasonable.

Given that mental health providers made the majority of IDDs, educational efforts to increase the awareness of the symptoms and presentation of bipolar disorder would probably only minimally reduce the rate of IDDs. On the other hand, education about the high rates of IDDs and the risk factors associated with them may be more effective in this provider population. To be effective, an intervention needs to result in the current physician receiving and incorporating information about the past bipolar diagnosis or symptoms into the current diagnosis.

### Limitations

This research utilized administrative claims data, which enabled the unobtrusive observation of usual clinical practice for a large number of individuals, a necessary condition for this research. However, administrative claims data have limitations given that the data was collected for reimbursement rather than research purposes. As a result, the measures in the data are not ideal, and the study design is limited to statistical rather than experimental control, leaving the data open to alternative explanations.

The diagnostic information in claims data may not be reliable. For some conditions, such as Alzheimer's disease [26] and myocardial infarction [27], claims diagnosis algorithms have been found to have high agreement with medical charts; however, the predictive value of our algorithm from claims diagnoses for bipolar disorder has not been demonstrated. Unützer and colleagues [5] conducted a chart review of individuals identified as having bipolar disorder based on various criteria in administrative claims and reported using an unspecified standard that a "reasonable" number of individuals with at least 1 inpatient discharge diagnosis or outpatient diagnosis had evidence of bipolar disorder in his or her medical chart. Our algorithm, which was more restrictive than the simpler criteria studied by Unützer and colleagues, required at least 2 diagnoses from hospital or physician visits that did not have exclusionary diagnoses and, therefore, should be at least "reasonably" accurate. However, even if the diagnoses in the claims match those in the patients' charts, they still may not coincide with the diagnoses made based on the gold standard Structured Clinical Interview for DSM-IV (SCID). Nonetheless, we believe our study population is representative, and our results can be generalized to similar populations.

Throughout this article, we have referred to the depression diagnoses following bipolar diagnoses as *incongruent *diagnoses rather than *misdiagnoses*. This has been in recognition that the diagnoses given in the claims may not reflect the true gold standard SCID diagnoses. In a recent study examining SCID diagnoses in outpatients, Zimmerman and colleagues found that less than half (43.4%) of individuals reporting having been diagnosed with bipolar disorder met the SCID criteria for the disorder [28]. Interestingly, 30% of those that did meet the SCID diagnosis had not previously been diagnosed with bipolar disorder. These findings suggest that not only is bipolar often under-diagnosed it is also over-diagnosed. This raises the possibility that the IDD in our study may have been the correct diagnosis. However, given the pattern of resource utilization, we believe that the bipolar disorder diagnosis was more likely to correct on average. Previous research in private payer claims has found that bipolar disorder is more costly than unipolar depression, particularly in terms of psychotropic medication and psychiatric hospitalization costs [29]. If the unipolar depression diagnoses had been correct more often than the bipolar, we would have anticipated the IDD group to have lower, instead of higher, resource use, particularly for psychiatric hospitalization.

One potential alternative explanation for our results is individuals who received the IDD following a bipolar diagnosis were simply more complex patients who, to no surprise, incurred higher costs. In the analysis, we utilized predicted probabilities to statistically control for background differences between the IDD and NIDD patients. A large number of baseline variables were used to construct the predicted probabilities (Table 1). From a theoretical perspective, because these variables were used to calculate the predicted probabilities and the analysis adjusted for predicted probabilities, the difference between the IDD and NIDD could not have been driven by these background differences [19]. To the extent that these background variables, including costs, comorbidities, and resource use, capture patient "complexity", we have ruled out this as a driver of the result. However, if another confounding variable exists that was not included in the predicted probability calculation, it could still explain our results. A study of an intervention in which administrative claims are screened and physicians are contacted when they file a claim with an IDD is needed to validate our results and accurately assess the cost savings that could be realized.

## Conclusions

An incongruent diagnosis of unipolar depression in persons previously identified with bipolar disorder appears to be relatively frequent and costly. Patients who received IDDs had increased psychiatric hospitalizations, ER visits, and ambulatory services. The apparent misdiagnosis may have resulted in patients not receiving the needed mood-stabilizing medications or receiving contraindicated antidepressant monotherapy. In this study, the IDDs appeared to arise when patients with bipolar disorder switch mental health providers, and the new provider may not be receiving information about past manic/hypomanic episodes needed to differentiate bipolar disorder from unipolar depression. This retrospective claims-based analysis needs to be validated with a prospective health management intervention study where an intervention occurs when an IDD is given to an individual who was historically diagnosed with bipolar disorder by a different provider. An effective intervention that informs a physician who submits a claim with a depression diagnosis for a patient about the patient's previous treatment for bipolar disorder could potentially improve patient care and save, on average, $1641 per patient per year in a managed-care population.

## Competing interests

Funding for this project was provided by Eli Lilly and Company (Indianapolis, Indiana, USA), including the article-processing charge. Michael Stensland was a full-time employee of Eli Lilly and Company and a minor stockholder while developing this research. Jennifer Schultz received funding from i3 Innovus to conduct the data analysis and serves as a consultant for the organization. Jennifer Frytak is an employee of i3 Innovus.

## Authors' contributions

MDS was involved with designing the study; interpreting the data; and manuscript writing and reviewing. JSS had full access to all the data in the study, completed the data analysis, and was involved with writing and reviewing of the manuscript. JRF was involved with designing the study, study implementation, interpreting the data, and reviewing the manuscript. All authors have read and approved the final manuscript.

## Pre-publication history

The pre-publication history for this paper can be accessed here:

http://www.biomedcentral.com/1471-244X/10/39/prepub
